# Abstinence-based treatment of comorbid eating disorders and ultra-processed food addiction

**DOI:** 10.3389/fpsyt.2025.1586490

**Published:** 2025-07-08

**Authors:** Susan Peirce Thompson, M. Joy Jacobs, David A. Wiss

**Affiliations:** ^1^ Department of Brain and Cognitive Sciences, University of Rochester, Rochester, NY, United States; ^2^ Bright Line Eating Solutions, LLC, Rochester, NY, United States; ^3^ Department of Psychiatry, School of Medicine, University of California at San Diego, San Diego, United States; ^4^ Dr. Joy Jacobs Inc, Los Angeles, CA, United States; ^5^ Nutrition in Recovery LLC, Los Angeles, CA, United States

**Keywords:** eating disorder (ED), ultra processed food addiction, abstinence based treatment, abstinence based treatment in food addiction, food addiction abstinence or harm reduction, food addiction intervention, eating disorder treatment

## Abstract

While not officially recognized as a clinical diagnosis, ultra-processed food addiction (UPFA) is an increasingly observed phenomenon that frequently co-occurs with eating disorders (EDs). Yet, treatment remains both understudied and controversial. Many challenges exist when treating patients with comorbid UPFA/ED, particularly in the context of the polarizing debate between abstinence-based and moderation-based approaches to nutrition intervention. We present three vignettes illustrating diverse trajectories of recovery when an abstinence-based approach is explored by a patient presenting with ED symptoms. Ultimately, some patients will recover with abstinence, while others may be harmed and fare better with a moderation-based approach. This dichotomy appears difficult for many patients and clinicians to navigate, particularly since integrative middle-ground approaches remain less characterized. Patients deserve individualized treatment plans from open-minded, experienced clinicians who can comprehensively assess genetic vulnerability; upbringing; and current neurobiological, psychological, and social/cultural presentation. We argue for a nuanced, multidisciplinary approach that may combine elements of both abstinence and moderation, tailored to the patient’s specific needs, emphasizing the importance of cross-disciplinary collaboration. More research is needed to develop evidence-based, patient-centered treatment options for UPFA in the context of other food- and body-related pathology.

## Introduction

In recent years, clinicians and researchers have posited that treatment approaches for individuals with certain types of eating disorders (EDs) should recognize that ultra-processed food addiction (UPFA) is a valid phenomenon and that abstaining from certain foods and behaviors may be helpful and necessary for some to achieve recovery (1, 2). Within the field of ED treatment, however, the construct of UPFA remains controversial, as many assert that addiction-like eating is merely a relic of restrained eating (3, 4). Despite the growing body of research over the last two decades, gaps remain, and UPFA has not been formally recognized as a clinical condition by the World Health Organization or the American Psychiatric Association.

Between 2010 and 2018, significant scholarly debate transpired in the published literature, with some arguing that UPFA or food addiction is a valid and helpful construct (5–8), with others questioning its validity and utility (9–12). Data that challenges UPFA suggest that people living at a body weight less than 10% below their highest adult weight and people with disordered eating behaviors manifest significant attentional bias toward high-calorie food cues and other cognitive and hormonal adaptations that may be underlying addictive-like eating (13–15). Thus, it became generally accepted in the ED field that addiction-like eating symptoms are best conceptualized as the results of weight suppression and dietary restraint and are not the manifestation of a substance use disorder (SUD).

Over the past seven years, scholarly articles have largely stopped challenging the existence of UPFA, and articles describing the construct continue to be published at a rate now exceeding 350 papers annually (16–18). Meanwhile, there is a noticeable paucity of randomized controlled trials and meta-analyses. Increasing scientific attention may reflect the noted utility of the framework, sociocultural trends, commercial momentum toward treatment, and/or the clinical and scientific interest in understanding aberrant eating patterns not adequately accounted for by traditional ED models. Recently, a global coalition of doctors, clinicians, and scientists (not randomly selected) united to engage in a year-long Delphi process to distill and then formally articulate a consensus, agreed upon by 37 out of the 40 participants (19). Their conclusions include the following: that sufficient research suggests that UPFA exists, that UPFA is the best name for the condition, that it can occur with or without a comorbid ED, that it should be listed in the ICD-11 as an SUD, and its symptoms are not fully accounted for by the current ED or obesity categories (19).

Many patients do present with comorbid UPFA and ED, and while the literature describing clinical support for overlapping EDs/UPFAs is notably sparse, this perspective piece calls for more cross-disciplinary collaboration to move the field forward.

The neurobiological evidence that ultra-processed foods (e.g., cookies, chips) impact both the mind and the brain similarly to drugs of misuse is substantial. Ultra-processed foods trigger similar dopamine (20–24), opioid (23–25), endocannabinoid (26, 27), and serotonin (28, 29) neurotransmitter downregulation (30), have similar patterns of cue reactivity in the brain (31–34), and are associated with cognitive impairments similar to those found with other misused substances (35). While ultra-processing appears to be the primary factor impacting which foods may confer addiction risk, some minimally processed foods high in fat and sodium, such as cheese, nuts, and bacon, also rank relatively high in addiction susceptibility (36). Meanwhile, there is not consensus on the precise addictive agent in food that drives the hedonic eating response, because it is generally a combination of refined carbohydrates, refined fats/oils, and added sodium. While the evidence for sugar addiction is substantial (37–43) and overall patterns suggest that ultra-processed foods drive addiction more so than minimally processed foods (36), more research is needed to determine the boundaries and scope of the addictive substances in the food supply. Without this clarity, some argue that an operational definition of UPFA as an SUD remains elusive. However, similar to SUD, susceptible individuals tend to develop dysfunctional relationships to the substances most frequently used to self-medicate unresolved distress.

Some authors have argued that UPFA is a misnomer and “eating addiction” is the better descriptor due to limited evidence for addiction to any of the three macronutrients (carbohydrates, protein, fat) (44). Others have shown that susceptible individuals describe loss of control over ultra-processed foods (e.g., ice cream, pizza) that represent hyperpalatable combinations of refined macronutrients plus processed culinary ingredients (e.g., sugar, salt), but not over minimally processed foods (e.g., legumes, vegetables) (36). We agree with the recent international consensus statement (19) and argue that the underlying issue is best conceptualized as substance-related rather than a generalized addiction to the process/behavior of eating, although both can present.

While both UPFA and ED typically involve common manifestations of disordered eating (e.g., loss of control, efforts to control), it is the characterization of UPFA as an SUD that distinguishes the conditions. Specific behaviors of binge eating, food restriction, and/or purging are hallmarks of ED, yet UPFA can (and often does) exist without any of those behaviors presenting. Thus, UPFA is operationalized by the same DSM-5 symptoms as other substance-related disorders, for example, using more than intended, experiencing cravings, a persistent desire to cut back but repeated failed attempts to do so, and continued use despite negative consequences (45, 46).

The most noteworthy gap in the literature concerns UPFA treatment (47), although studies and scholarship exist (48–50). Despite this gap, treatment options have been around for decades, including inpatient treatment (51, 52), online and in-person intensive programs for people with UPFA (with or without ED) (53), online and in-person peer-support and self-help (i.e., 12-Step) programs (54), as well as abstinence-based food addiction treatment for weight management with ED support (55, 56). A training program based on guidelines from the American Society for Addiction Medicine graduates clinicians to become Certified Food Addiction Professionals in Europe and the United States (57).

ED clinicians who recognize addiction-like eating in some of their patients may choose to assess whether UPFA is present and, if clinically appropriate, offer individualized treatment approaches (58). But what is meant by “abstinence” when it comes to food? Humans must eat to stay alive, but we do not need ultra-processed foods to live. Do some people fare better in recovery by strategically removing specific triggering foods? Unfortunately, in the contemporary food environment inundated with accessible and affordable ultra-processed foods, socially advantaged individuals tend to have more resources to explore this path. Treatment options that favor advantaged groups run the risk of widening health disparities. Thus, targeted research is needed to identify the feasibility of abstinence-based interventions among vulnerable populations.

In the context of UPFA, “abstinence” can be individualized but typically refers to either refraining from eating specific foods or ingredients (e.g., refined sugar, flour, alcohol, ultra-processed foods, and/or “personal binge foods”), analogous to abstaining from alcohol, caffeine, or opiates to recover from a SUD (36, 59); and/or refraining from behaviors like snacking, grazing, or overeating (e.g., eating only at mealtimes, weighing and measuring portions) to treat the “process addiction” related to food consumption (44, 60, 61).

An abstinence-based approach to UPFA has been proposed in the scientific literature (16, 48), and a few preliminary studies have tested its efficacy, suggesting positive results (62–64). A weight neutral harm reduction approach to addictive eating demonstrated improvements in disordered eating behaviors with no signs of adverse changes (65). More aggressive interventions have implemented a ketogenic version of an abstinence-based food plan. There is a biological rationale for the use of a ketogenic diet in treating psychiatric conditions in general, and, surprisingly to some, case reports of remission from anorexia nervosa have been reported (66). Other studies examining the results of abstinence from refined sugar, flour, and ultra-processed foods with a balanced macronutrient diet have been published in the context of weight management (55, 56, 67).

12-Step programs have historically formed the foundation for many abstinence-based frameworks for recovery from various addictions, spawning many programs related to food (55, 68). Most of them advocate for some form of an abstinence-based approach to treating food issues. Still, little research exists examining the effectiveness of these programs, as they are designed to be anonymous and non-clinical (69, 70). Abstinence-based approaches to other SUDs, including detoxification programs, outpatient programs, residential rehabilitation programs, 12-Step programs, and therapeutic communities, have been extensively studied, including in comparison to harm-reduction approaches (69–75). For SUD, complete abstinence has been found more effective than conditional abstinence or harm reduction (70, 75) but is not always feasible. Long-term abstinence from ultra-processed foods has never been documented, but thousands of clinical anecdotes exist. Some have suggested that abstinence-based approaches could appeal to those with restrictive EDs or even confer risk for the development of restrictive EDs, such as anorexia nervosa (76). In the ED treatment field, many assume UPFA recovery to be synonymous with 12-Step, since they have a rich history and form the foundation of some clinical philosophies. However, much like SUD recovery, approaches to recovery have expanded beyond 12-Step models to capture a broader range of clinical presentations (76).

The alternative to abstinence-based treatment is moderation or harm reduction. This approach generally encourages “all foods fit” (1) and eschews food rules, limitations, or restrictions. Since many patients with EDs have developed a host of food rules that scaffold their disordered eating, it makes sense that a psychologically-based treatment modality would aim to dismantle or reduce over-adherence to food rules. One criticism is that many ED clinics and treatment professionals ignore the construct of UPFA because its implications do not fit well into this paradigm. Individualizing nutritional treatment can be challenging in a milieu and for a food service operation.

When it comes to the nutritional management of disordered eating, the abstinence vs. moderation approach has a history of conflict (1, 2, 4). In the ED community, the construct of UPFA is generally considered unhelpful, potentially leading to worsening of binge eating frequency and severity in patients, as well as increased fear of eating certain foods and a more negative self-concept, including internalized weight stigma (1, 4, 76). Among health professionals in general, one study found that 50% consider the term “food addiction” to be stigmatizing; 60% reported that they were interested or very interested in receiving training in food addiction; and 72% reported that their patients had asked them about addictive eating (77). This study did not, however, separately examine the views and experiences of different types of health professionals (e.g., nutritionists vs. psychologists vs. ED specialists vs. mental health generalists). We hypothesize that a higher percentage of ED clinicians would consider the term “food addiction” to be stigmatizing compared to SUD professionals, who generally view the underlying neurobiology as removing blame from the individual. For reasons of discipline bias and ongoing “culture wars,” the moderation approach is favored in most ED clinics (76).

Such polarizing views make sense when considering the selection bias inherent in the case presentation and treatment history that various patients and practitioners may encounter. For example, clinicians treating UPFA with abstinence-based approaches are likely to witness success stories of people who have benefited from that approach, and encounter people who have failed to find relief attempting to eat all foods in moderation. Meanwhile, professionals working in ED treatment will frequently encounter people who have not succeeded with abstinence and may have been harmed by it. Thus, a great divide exists because treatment providers see the fallout from the competing philosophy. Our clinical experience advocates for integrated rather than binary approaches. Some individuals presenting for ED treatment will not reach complete remission using moderation-based food plans (perhaps related to a host of factors, not just the food) (78). This has led some investigators to hypothesize that testing for UPFA and including abstinence-based or low-exposure options may improve recovery rates (79).

There is a middle ground that is nuanced, complex, and requires individualized assessment from professionals trained in classic ED presentations and SUD neurobiology. As mentioned, it remains challenging to individualize nutrition treatment in large-scale food service operations and treatment milieus with mixed case presentations. What is medicine for one person could be poison for another, and the dose matters. Some individuals are likely to benefit from any therapeutic approach, be it moderation, targeted abstinence from specific foods, a robust program of abstinence, GLP-1 medications, or complementary and alternative methods such as acupuncture, provided they get more support than they currently have (80–86). Furthermore, what works in one life period may not work indefinitely. Therefore, one snapshot in time cannot tell the whole story. Experienced, open-minded clinicians who conceptualize a timeline, use a battery of assessment tools, and are familiar with multiple frameworks are needed to help patients find meaningful and sustainable recovery (86). Multidisciplinary collaboration is critical for recovery from disordered eating, and it seems imperative that professionals do not confuse patients with conflicting (or opposing) philosophies (2, 58). Reducing clinician bias can be additionally challenging if the provider has lived experience with recovery that influences their treatment philosophy.

It is our goal to address the apparent tension between proponents of moderation-based and abstinence-based approaches to ED treatment. Toward that aim, we present three vignettes, based loosely on patterns observed over the years in our professional work, illustrating ways that an abstinence-based treatment approach can become attractive to patients with disordered eating, and the varying degrees of success utilizing such abstinence-based approaches. Importantly, it should be noted that these vignettes illustrate trajectories we have seen play out repeatedly in our collective experience, but should not be interpreted as empirical evidence to support a specific treatment model. Rather, they are a springboard for discussion and reflection among clinicians facing increasingly complex cases where UPFA can underly an ED presentation. These vignettes are broadly based on individual people, but details have been generalized to illustrate patterns common to treatment seekers (see **Table 1**).

**Table 1:** Summary demographics of three illustrative vignettes, based loosely on patterns we have observed repeatedly in clinical practice.

Vignette:	#1	#2	#3
Alias	“Ryan”	“Mallory”	“Solana”
Age:	64	36	49
Gender:	Male	Female	Female
Race/Ethnicity:	Mixed Race	Caucasian	Hispanic
Marital Status:	Single; Divorced	Married	Married
Occupation	Retired from the nonprofit sector	Psychotherapist	Attorney, not currently practicing
Height:	5 feet 11 inches	5 feet 8 inches	5 feet 7 inches
Current Weight:	175 lbs.	143 lbs.	160 lbs.
Highest Adult Weight:	290 lbs.	160 lbs.	176 lbs.
Lowest Adult Weight:	150 lbs.	115 lbs.	125 lbs.
Comorbid Diagnoses:	Generalized Anxiety Disorder, Substance Use Disorder, Post-Traumatic Stress Disorder, Major Depressive Disorder	Generalized Anxiety Disorder, Major Depressive Disorder	Type 1 Diabetes

## Vignette 1

Ryan was the oldest of seven siblings. Growing up, there were many relocations and many babysitters, one of whom sexually abused Ryan when he was 11. His parents drank frequently and often belittled him verbally. Ryan ate to cope and gained significant weight during his early teen years. He was teased for his weight and pretended that it did not bother him, but it did.

Ryan discovered an escape through alcohol and drugs in high school. Those substances allowed him to start deprioritizing food, and he lost weight, which was positively reinforcing. He started binge eating in college, followed by multiple days of fasting to compensate. Over the following decades, he tried various diets, binged, restricted food intake, consumed various laxatives and diuretics, and continued to drink alcohol and use illicit drugs. He attended several inpatient ED clinics, completing some and leaving others early. Every time he ate sweets (e.g., pastries), he experienced loss of control in binge-like patterns. His weight cycled but trended upward, and physicians warned him of looming health problems.

Ryan got clean and sober in 2015, but subsequently, his bingeing worsened. In 2017, he took an assessment that revealed that he met criteria for a food addiction, so he enrolled in an abstinence-based group online program. After an initial period of success, his bingeing and restricting behavior reemerged and continued for another year. Ryan sought more help, joining a small group coaching program and getting individualized support tailored to address his food addiction and disordered eating conjointly. He quickly found freedom using an abstinence-based food plan along with social support and has been abstinent from binge eating and food restriction since late 2018. Since that time, his days of poor mental health have decreased significantly, and he now reports an overall feeling of flourishing, rather than just surviving.

## Vignette 2

Mallory was one of five children who grew up in a chaotic household. Her parents eventually divorced, and Mallory lived with her siblings, codependent mother, and controlling stepfather. She was relieved to leave home at age 18.

Mallory reports a lifelong heightened sensitivity to the size and shape of her body. Although in a normal weight range, she began dieting in high school. It progressed to a clinically significant ED when she felt lonely, anxious, and depressed, living on her own at age 19. She restricted her food intake aggressively and lost her menstrual cycle. This period of anorexia nervosa lasted over two years and transitioned to bulimia nervosa (vomiting). Mallory struggled with bulimic symptoms for 12 years, with only short periods of remission from purging behaviors.

To find recovery, Mallory attended Overeaters Anonymous, individual psychotherapy, and an ED-focused intensive outpatient program (IOP). The IOP helped her recover from bulimia, and her ED stayed in remission for many years.

Mallory explored an abstinence-based approach to eating when she found a group that assisted those with food addiction using an abstinence-based approach. She joined because she wanted to lose 10 lbs., despite the warning from her ED therapist. Abstaining from refined sugar and flour triggered her restrictive ED mindset, and she relapsed into bulimia. After seeking support and returning to an approach to eating that included eating all foods in moderation, the bulimia remitted, and she now knows that an abstinence-based approach to eating does not work for her. She has regained significant peace and freedom with her food and her body, adhering to a balanced approach with unconditional permission to eat and enjoy all foods.

## Vignette 3

Solana was the second of four children. She grew up in a small, rural town where they were among the only ethnic minority members of the community. Her father was a “functional” alcoholic, and her mother, an attorney, was a consistent, loving presence in her life.

Solana overate as a child. She went on her first diet at age 15, restricting her intake to 1000 calories or less per day. She lost weight rapidly, and her mother expressed concern. Shortly thereafter, she had her first binge, and bingeing continued through her teen years.

At age 21, Solana was diagnosed with Type 1 diabetes. Her eating felt out of control; she felt like she had no “off button” for her food intake. She binged and gained weight, engaging in multiple failed attempts to control her eating and/or lose weight over the subsequent two decades. She secretly learned to manipulate her insulin to “manage” the impact of her binges.

Solana joined an abstinence-based food program in 2020 and lost about 20 pounds within a few months. But for the next four years, Solana struggled to maintain her abstinence, experiencing frequent binge episodes. During that time, she intermittently experimented with returning to a more inclusive moderation-based approach to eating. No matter what she did, she continued to binge, even when her life circumstances were favorable.

After much experimentation, Solana found an individual therapist with a clinical practice treating concurrent EDs and UPFA. She got evaluated for food addiction and returned to an abstinence-based approach, this time adding more food and more consistently balanced meals to her food plan, and her binge eating fully remitted. Solana had to come to terms with the fact that her binge eating would only remit at higher body weights and did the necessary body image work to stay in recovery. She now feels free of the food obsession but often fantasizes about weight loss; however, she does not act on it. She reports finally growing into the woman she always imagined she could be.

## Discussion

Our three vignettes elucidate that there is not one universal trajectory when someone with an ED history adopts an abstinence-based treatment program. For some, like Ryan and Solana, UPFA is comorbid with ED symptoms, and a treatment approach that encourages eating foods like cake and fast food “in moderation” will likely result in continued relapses and/or progression of symptoms. For patients like these, an abstinence-based approach may be the best path for long-term symptom remission, independent of weight status. However, for other patients, like Mallory, UPFA symptoms are secondary to ED, and moderation is more effective for recovery. For those with a restrictive mindset and a lot of anxiety related to body image, an abstinence-based approach to eating can exacerbate symptoms.

There is also a middle-ground approach that involves selective abstinence from some foods or eating behaviors (e.g., strict avoidance of one or two specific binge foods that cause the most distress) combined with a harm reduction or moderation approach to other foods (4). Many patients prefer this lower exposure option because it is more inclusive, flexible, and feasible. Some will find that it can work long-term, provided they continue to assess problematic foods and be honest with themselves and their support network. Others may find inclusive eating a helpful stepping-stone to “all foods fit” or, alternatively, toward a more clearly defined abstinence-based approach. The recovery trajectory is likely to evolve as life circumstances change, and it is critical to have a network of supportive people who understand the nuance and will not superimpose their definition of recovery.

Critically, at any one point in time, it can be challenging for clinicians to discern what ultimately will work best. Thus, supervised experimentation can be part of the recovery process. People for whom abstinence is helpful in the short term can find that it activates binge or purge episodes at other times, as happened with both Ryan and Solana. Binge/purge episodes can also be a cue to abandon rigid abstinence and revert to inclusive eating, as with Mallory. Assessment tools can be helpful, but do not replace insight from professionals who can conceptualize a life history in the context of body image dissatisfaction, complex trauma, and other psychiatric traits. Administering the YFAS 2.0 (45) or conducting a food addiction clinical interview like the Food Addiction Symptom Inventory (87) can reveal whether UPFA is comorbid with the ED. However, interpreting these scores without understanding the underlying cognitive processes and context that drive over- and undereating might miss the mark.

If a patient is not successful with abstinence, it may indicate that it is an ineffective framework for them, but could also suggest they need more intensive support, psychoeducation, and guidance. Some treatment options, such as 12-Step groups or large online group programs, provide less intensive treatment but offer peer-to-peer support, which can be more affordable/accessible but often lack the nuanced approach (described herein) that cross-disciplinary professionals can have. It may be that the patient needs an inpatient treatment program, an outpatient intensive, a small group coaching program, or individual therapy. Trauma therapy is usually indicated, and without it, many individuals fail to achieve sustainable recovery (58).

Unfortunately, many of these options may not be accessible to people of lower socioeconomic status (SES), who may struggle to implement abstinence in environments where fresh, whole foods are less available or culturally less desirable. Food companies have a long history of targeting minoritized groups in their marketing strategies (88). Even in higher SES communities, patients striving for abstinence may be faced with over-access, advertisements, and social pressures to indulge. These obstacles are very real for most patients, and in part can be attributed to the fact that UPFA is not yet recognized as a mental health disorder, and so much confusion exists on this topic.

There is evidence for a genetic vulnerability to the development of UPFA, which should be assessed in clinical intake (89). Note that both Ryan and Solana have alcoholism in their family. While a family history of alcoholism/addiction is not deterministic of offspring addiction, it can be used as a clue to investigate whether UPFA may be underlying disordered eating behaviors. An experienced clinician should use assessment tools to explore whether UPFA is present when a family history of addiction exists.

It has been suggested that false positives can exist when assessing for UPFA (58). If an individual has been dieting, fasting, or otherwise restricting food intake and/or has recently lost significant weight, they may screen positive for UPFA. This can be due to psychological processes (e.g., perceived lack of control) or metabolic changes in hunger and satiety hormones that follow significant weight loss or food restriction (90). A skilled clinician will conduct a comprehensive case history and, with a positive UPFA screen following food restriction or weight loss, aim to stabilize their weight, then readminister the addiction assessment after homeostasis has been reached. More research is needed to develop evidence-based clinical protocols to guide clinicians in applying this type of stabilization procedure to avoid false positives in the assessment of UPFA.

We humbly acknowledge that, despite global consensus among clinicians and researchers recognized as food addiction experts, within the field of ED treatment, the construct of UPFA is still highly controversial. As emphasized, abstinence-based therapies can be harmful for some ED patients and lead to worsening of binge eating symptoms, increased preoccupation with food, rigidity around food, binge-restrict cycling, and heightened food fears. Such ethical considerations may be one reason there is a shortage of clinical trials. Mallory’s experience highlights this concern—her symptomatology got worse, not better, when she stopped eating refined sugars and flours. These realities create understandable backlash in the ED community when clinicians propose the use of abstinence-based treatment protocols. However, one could argue that the encouragement to regularly eat desserts and other ultra-processed foods among those with true positive UPFA can lead to similar ethical conundrums.

Working with treatment-resistant ED is not straightforward. Although abstinence-based food plans may challenge entrenched philosophies and make some treatment providers uncomfortable, their efficacy cannot be ignored when appropriately applied. There is a missed opportunity to help people who aren’t getting better. As scientists and clinicians, we have a duty to stratify our patient population based on the available evidence to correctly identify potentially promising interventions rather than apply broad strokes in scalable treatment models.

Undoubtedly, more research is needed on alternative treatment options for patients who present with ED symptoms along with addiction-like eating. It appears that 12-Step approaches have fallen out of favor in the current weight-neutral ED paradigm, and newer approaches that tackle issues of internalized weight stigma are needed. Meanwhile, the evidence that UPFA exists and is impacting a subset of our clients with disordered eating is too significant to be ignored (1, 4). Skilled clinicians with backgrounds in both SUD and ED can take the lead in understanding the complexity of UPFA and learning how to question long-held assumptions when evaluating patients. Along this line, developing clinical experience with the wide range of trajectories that may unfold when UPFA is treated comorbidly with an ED will offer new possibilities for patients seeking (and deserving) a nuanced, supportive treatment approach.

## Data Availability

The original contributions presented in the study are included in the article/supplementary material. Further inquiries can be directed to the corresponding author.
